# Quantifying experimental errors in measuring colloidal interaction potentials with optical tweezers

**DOI:** 10.1039/d5sm00551e

**Published:** 2025-09-01

**Authors:** José Muñetón-Díaz, Augustin Muster, Luis S. Froufe-Pérez, Frank Scheffold, Chi Zhang

**Affiliations:** a Department of Physics, University of Fribourg 1700 Fribourg Switzerland frank.scheffold@unifr.ch chi.zhang2@unifr.ch

## Abstract

We present a systematic study on the measurement of pair interaction potentials between small particles using optical tweezers (OT), focusing on the modelling and quantification of three key experimental errors: *z*-motion error, dynamic error, and static error. While these errors have been previously acknowledged, their individual effects on such measurements had not been thoroughly examined. We develop a framework to model these errors and validate it through controlled experiments. By carefully tuning experimental parameters, we decouple and quantify each error source, demonstrating that they can be independently controlled and accounted for. Our approach enables a more precise access to the true interaction potential, reducing measurement ambiguities and improving the accuracy of comparisons with theoretical models. As a demonstration of the framework's applicability, we apply our correction method to extract the depletion attraction potential from experimentally measured data, showcasing how systematic error removal enables the retrieval of physically meaningful interaction potentials. This work provides a robust methodology for enhancing the accuracy of OT-based potential measurements and for studying colloidal interactions.

## Abstract 2

We experimentally quantify the key error sources in optical tweezer measurements and systematically model their impact on the extraction of interaction potentials.

## Graphical abstract

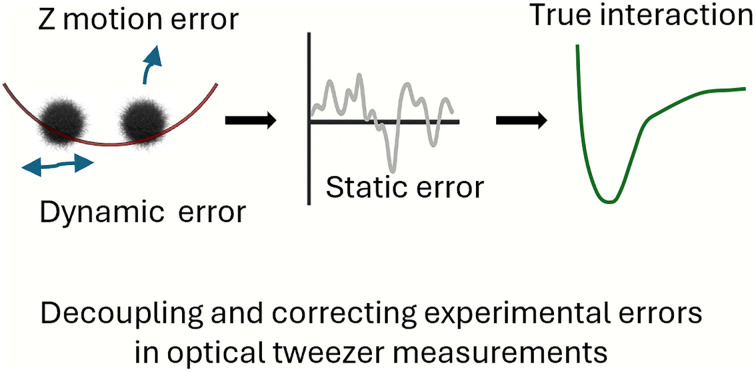

## 1 Introduction

Interaction potentials between colloidal particles are fundamental to understanding phase transitions, self-assembly, and emergent properties in complex fluids, soft matter, and biological systems.^1–5^ Precise measurements provide deeper insights into the stability, phase behaviour, and structural transformations of colloidal systems, facilitating the development of theoretical and predictive models.^6–8^ Researchers have inferred interaction potentials through various methods, including the analysis of colloidal phase behaviour and equations of state,^9,10^ inversion of pair-correlation functions *via* liquid-state theories,^11,12^ and direct force measurements using techniques such as the surface force apparatus and atomic force microscopy (AFM).^13,14^ However, these methods either measure interactions indirectly or involve at least one macroscopic surface. To directly measure interactions between two (or more) colloidal particles, researchers have developed alternative experimental approaches, with one of the most direct being optical tweezers (OT).^15–19^ In the latter approach, two colloidal particles are trapped along a laser-generated optical line, or alternatively in two point-traps, and their centre-to-centre distance fluctuations provide a direct way to determine their interaction potential.

Although OT-based measurements offer high precision, obtaining an accurate interaction potential from experimental data is still challenging because of unavoidable experimental errors. The first major source of error arises from out-of-plane (*z*-motion) fluctuations.^20,21^ Optical traps often operate in a two-dimensional imaging plane, but particles move in three dimensions. The limited trap stiffness leads to axial displacements, which distort the measured 2D interparticle distances. The second error source is a dynamic error, which results from position blurring due to the finite exposure time of the camera. Since the recorded particle positions are effectively time-averaged over the exposure time, systematic biases emerge in the measured particle separations.^22–24^ The third source of error is a static error, which originates from localisation uncertainty due to imaging noise and finite resolution, blurring the extracted pair distances.^23,24^

Previous studies have acknowledged these sources of errors, often treating them collectively as a single uncertainty contribution.^25,26^ In our previous work,^27^ the influences of dynamic error and static error were discussed. However, their individual effects on measured potentials have not been systematically examined. This lack of clearly separating the different contributions can obscure the role of each error type, leading to potential biases in the extracted interaction potentials. An incomplete understanding of these contributions limits the precision and reliability of experimental results.

In this work, we develop a systematic approach to model, quantify, and correct the effects of experimental errors in OT-based potential measurements. By independently tuning experimental parameters, we isolate the influence of the *z*-motion error, dynamic error, and static error, allowing their individual contributions to be quantified. Our study provides a clear guideline for performing accurate potential measurements using optical tweezers by identifying key sources of errors and outlining methods to mitigate their impact. The framework we establish enables the correction of these errors, significantly improving the precision of extracted interaction potentials. This advancement represents a major step toward achieving absolute potential measurements, where systematic distortions are minimised, and the true interaction potential can be reliably determined. To demonstrate the effectiveness of our approach, we apply our correction method to extract the depletion attraction potential from experimentally measured data, showcasing how error removal enables the retrieval of physically meaningful interaction potentials.

All measurements in this study were conducted using line optical tweezers (LOT). However, the methods and modelling presented are equally applicable to measurements performed with multi-point tweezers.

## 2 Methods

### Origin and modelling of the experimental errors

**Fig. 1:**
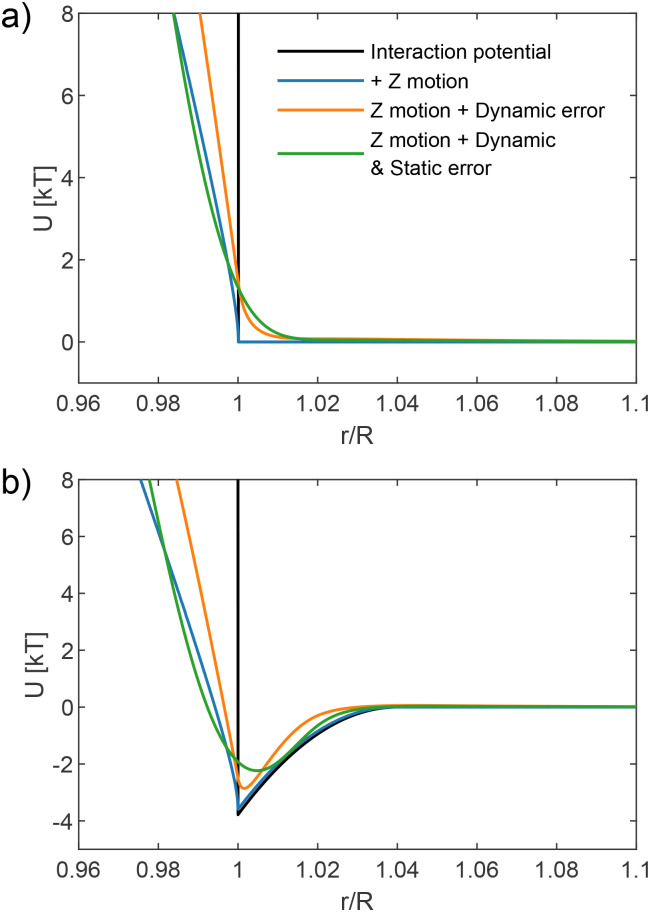
Modelling the influence of experimental errors on the measurement of interaction potentials. We begin with an initial potential—either a hard-sphere repulsion (a black line in a) or a typical depletion attraction (a black line in b) of depth 4*k*_B_*T*. The first error introduced is due to the out-of-plane (*z*-direction) motion, modelled using a Gaussian displacement with standard deviation *σ*_*z*_. This effect causes the potential to bend toward shorter distances, particularly in regions of strong repulsion (≫*k*_B_*T*). Next, a dynamic error is added, accounting for the time-averaging during finite camera exposure time and characterised by *σ*_D_. Finally, a static error—associated with localisation uncertainty in particle tracking—is added, described by *σ*_S_. After all three sources of errors are taken into account, the resulting potential curve (shown in green) can be directly compared to experimental data. The parameters *σ*_*z*_, *σ*_D_, and *σ*_S_ serve as tunable parameters that quantify the influence of experimental uncertainties in the model. In both panels, *σ*_*z*_ = 0.05*R*, *σ*_D_ = 0.01*R*, and *σ*_S_ = 0.005*R*, reflecting uncertainties encountered under typical experimental conditions.

To ensure reliable extraction of interaction potentials, it is necessary to understand and quantify different experimental errors. In this section, we present a theoretical framework to model the three primary sources of errors—the *z*-motion error, dynamic error, and static error—before experimentally verifying their contribution. The error due to *z*-motion arises because the measured centre-to-centre distance from a 2D (top-down) observation is different from the actual 3D separation – due to the out-of-plane motion.^20,21^ The dynamic error originates from the finite exposure time: the recorded “snapshot” is, in fact, time averaged over the exposure time interval.^22–24^ Therefore, the particle position we extract from the image is also averaged. Finally, the static error results from the limited precision of particle tracking, which is governed by experimental noise when an image is recorded.^23,24^ Each error contribution arises at a different stage during the experiment: the *z*-motion error during the particle movement, the dynamic error during image recording, and the static error during image analysis. To accurately mirror these stages, our error-modelling process follows the same order, as illustrated in Fig. 1. We begin by considering a true interaction potential *U*(*r*) (in units of *k*_B_*T*) as a function of the centre-to-centre distance in three dimensions. We selected two example potentials to discuss the influence of the various sources of errors: the black line in (a) represents a hard-sphere potential, while in (b) it represents a depletion attraction potential well of depth 4 *k*_B_*T*.

We model the *z*-motion with a Gaussian distribution function *Q*_*z*_(*z*) = *N*[0,*σ*_*z*_^2^], which describes the probability of the particle being displaced by a distance *z*. When a particle moves out of plane, the true 3D separation is given by 
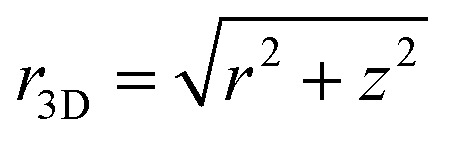
, where *r* is the observed 2D distance. The observed potential is determined by averaging over all possible axial displacements. The probability of observing a given *r* is weighted using the Boltzmann factor *e*^−*U*(*r*)^:1
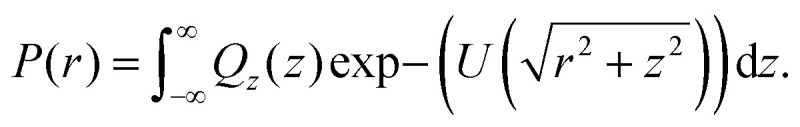
where *P*(*r*) represents the 2D distribution of interparticle distances, which would be observed if particle positions were sampled instantaneously.

In practice, however, each image is captured over a finite exposure time, during which the particles undergo Brownian motion. This results in a time-averaged recorded distance rather than an instantaneous one. To account for this, we model the recorded position as an average over fluctuations that occur during the exposure time. Let us assume that at the start of an exposure, the particle pair is at a distance *r* (which corresponds to the value measured at zero exposure time). During the exposure time, thermal motion causes their separation to fluctuate continuously. The recorded distance is the average of this fluctuating trajectory and differs from the starting distance by a deviation *a*, such that the recorded distance is *r* + *a*. The probability of observing a given averaged deviation *a* from position *r* is described by the distribution *Q*_D_(*r*,*a*). In the case of purely diffusive motion, *a* follows a Gaussian distribution *N*[0,*σ*_D_^2^] (see the SI Material), where *σ*_D_ represents the characteristic range of fluctuations during the exposure. In the absence of interactions, these fluctuations have no directional bias (*i.e. a* can take positive and negative values) and are independent of *r*.

However, in the presence of an interaction potential, the energy landscape biases the motion. Fluctuations that move the system to lower potential energy are favoured, while those that increase energy are suppressed. To account for this, we include an energetic weighting into the fluctuation model. The probability of a fluctuation from *r* to *r* + *a* is given by:2
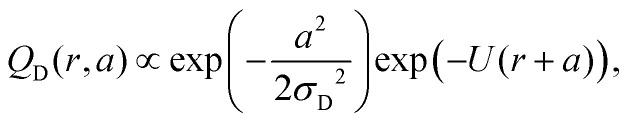
where *U*(*r* + *a*), the potential at the recorded position, modulates the probability of such a fluctuation that drifts the particle from position *r* to the recorded position *r* + *a*. When *U*(*r*) is flat, *Q*_D_ reduces to a pure Gaussian. In regions of strong confinement or attraction, the Boltzmann term suppresses motion, effectively reducing the dynamic error.

The probability of observing a recorded distance *r* is obtained by summing over all possible fluctuations *a*, each weighted by the probability that the particles were initially at *r* − *a* and then experienced an average deviation of *a* during the exposure time.3
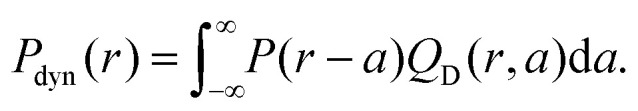


In the final step, we account for the static error introduced by localisation uncertainty, *s*, in particle tracking. This error arises from factors such as image noise and it leads to an additional blurring of the measured interparticle distances. We model this effect by convolving the dynamically corrected distribution *P*_dyn_(*r*) with a Gaussian kernel, *Q*_S_(*s*) = *N*[0,*σ*_S_^2^], where *σ*_S_ characterises the standard deviation of the tracking uncertainty.4
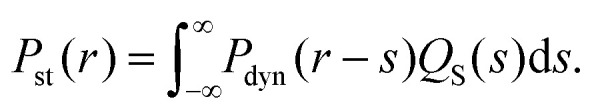


This final convolution completes the modelling of experimental distortions in the measured interparticle potential. The resulting distribution can then be converted into an effective interaction potential using the inverse Boltzmann relationship: *U*_modelled_(*r*) = −ln *P*_st_(*r*). Fig. 1 illustrates, step by step, how each experimental error modifies the measured potential, starting from the ideal true interaction.

### Experimental setup

To experimentally validate the effects of the modelled errors, we employ a line optical tweezer (LOT) system to trap colloidal particles. A 1064 nm fibre laser (YLR-10-LP, IPG Photonics) is used to create the optical trap in the focal plane of a Nikon Ti2 inverted microscope. The system employs a 100× APO TIRF objective with a numerical aperture (NA) of 1.49 to achieve tight optical confinement. Two distinct experimental configurations are used: an acousto-optic modulator (AOM)-based setup for time-shared traps and a spatial light modulator (SLM)-based setup for static traps.

In the AOM-based setup, the laser is directed through an acousto-optic modulator (DTSXY-400-1064, AA Opto-Electronic) before being expanded and collimated into the back aperture of the objective. A function generator (Tektronix AFG3022C) supplies a designed waveform to the AOM, causing the focal spot to scan rapidly over a length of approximately 4 μm at a frequency of 10 kHz, forming a Gaussian intensity profile. This scanning frequency is much faster than the timescales of Brownian motion, resulting in the formation of an effective time-averaged line trap. The laser polarisation is aligned parallel to the trap axis to minimise optical binding effects.^27^

In the SLM-based setup, a spatial light modulator (X10468, Hamamatsu) is used to modulate the wavefront of the trapping laser, creating a static line trap. A computer-generated hologram is applied to the SLM to shape the beam, with additional wavefront corrections implemented following the methods described in ref. 18 and 28.

Polystyrene (PS) particles (Bangs Labs, USA) of nominal radii of *R* = 250 ± 20 nm and *R* = 355 ± 20 nm are used in the experiments. We select particles of small size to highlight the influence of errors on the potential. Given our experimental setup, using even smaller particles would result in an unstable trap, leading to unreliable measurements. In most practical cases, as reported in the literature, slightly larger particles are more commonly used. We suspend the particles in a 5 mM KCl aqueous solution. Two particles are simultaneously trapped within the optical line trap, allowing for measurements of the interparticle potential. High-speed sCMOS cameras (Zyla Andor for the AOM setup and Prime 95B Teledyne Photometrics for the SLM setup) are used to record particle positions.

### Extraction of the intrinsic pair potential

The optical trap (OT) is used to position the particles, and it also induces optical binding forces. As shown in our earlier work^27^ and those of others,^26,29^ these optical forces can be described quantitatively. After removing the optical forces, we obtain what we refer to as the intrinsic potential, which is still affected by the errors discussed here—thereby concealing the true potential. We summarize the course of the data analysis as follow. The centre-to-centre distances between particles in the LOT are determined using a tracking method that fully reconstructs the images.^27^ Briefly, we first apply standard centroid tracking^30^ to provide an initial guess for the particle positions. The final, more accurate positions are then obtained by iteratively optimising both the particle positions and the shape parameters to best match the reconstructed image to the recorded intensity pattern. The probability distribution of these distances is then converted into a raw potential using the Boltzmann relation. This raw potential includes contributions from the optical trap potential *U*_OT_(*r*), the optical binding potential *U*_OB_(*r*), and the intrinsic pair potential *U*(*r*). As described in ref. 27, *U*_OT_(*r*) and *U*_OB_(*r*) are carefully removed, isolating the intrinsic pair potential between the particles.

It is important to note that the optical binding potential *U*_OB_(*r*) varies depending on the technique used to generate the line optical trap (LOT). Since the optical binding effects fundamentally arise from light scattering, the specific method by which a line trap is created influences the resulting optical binding potential. Time-shared LOTs, generated using acousto-optic modulators (AOM), and holographic-static LOTs, created using spatial light modulators (SLM), differ in their temporal and spatial distributions of the scattered optical field, leading to distinct optical binding interactions.

We investigate the differences in optical binding between these two setups, with both experimental measurements and calculations using discrete dipole approximation (DDA).^31^ The DDA results show excellent agreement with experimental data, as illustrated in Fig. S2, and their numerical stability is confirmed through a convergence study presented in Fig. S5. Further details on the optical binding effects are provided in the SI. The static LOT generates an optical binding interaction characterised by oscillations that extend over long distances, with a slow decay following an approximate 1/*r* scaling. In contrast, the time-shared LOT produces an optical binding potential with minimal oscillations, which decays more rapidly as the interparticle distance increases. In both cases, the optical binding force is most pronounced when the particles are in close proximity. In some cases, in static LOTs, the optical binding force can become so strong that it effectively prevents the particles from approaching each other, posing challenges for exploring the intrinsic pair potential in their specific regime. Conversely, the time-shared LOT can sometimes induce a strong attractive optical binding force, leading to excessive pushing or even permanent binding of the particles.

The choice of the LOT technique should take into account the particle size, material, and intrinsic interactions of interest. As an alternative approach to LOT and multi-point optical tweezers, blinking optical tweezers (BOT) temporarily switch off or rapidly modulate the traps, allowing particles to interact freely without optical binding forces or external trap potentials.^32^ This reduces experimental complexity and enables direct extraction of intrinsic pair potentials. However, the lack of confinement in BOT may lead to larger out-of-plane fluctuations, making it more challenging to accurately determine true 3D interparticle distances.

### Disentangling and quantifying errors in experiments

To isolate the influence of each source of error, we designed our experiments so that one parameter affecting a specific error type was varied while all others were held constant. The *z*-motion error is primarily affected by the depth at which the trap is located within the sample, as optical aberrations change with the focal plane position.^33,34^ The dynamic error is controlled by adjusting the exposure time of the camera, with longer exposure times leading to more pronounced time-averaging effects. The static error is influenced by the signal-to-noise ratio (SNR) of the recorded images, which is tuned by modifying the illumination intensity. For each case, the particle trajectories are recorded and analysed to quantify deviations caused by each error type. These experiments are used to validate the modelling process, leading to correction methods that allow us to recover a more accurate representation of the true interparticle potential.

**Fig. 2:**
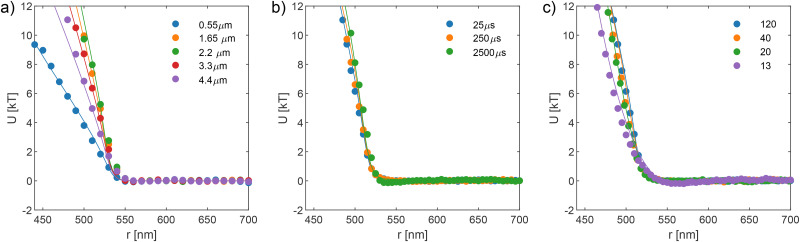
Influence of the *z*-motion error (a), dynamic error (b), and static error (c) on experimentally extracted pair potentials between polystyrene particles with a radius of 250 nm. Panel (a) shows the impact of the *z*-motion error by varying the trapping depth in the sample from *z* = 0.55 μm to *z* = 4.4 μm. Solid lines: measured interaction potentials were modelled using a hard-sphere potential with optimal settings of *σ*_D_ = 5 nm and *σ*_S_ = 3 nm, and we find *σ*_*z*_ values of 76, 46, 42, 50, and 58 nm (top to bottom in the legend). Panels (b) and (c) present measurements taken at an optimal depth of 2.2 μm and a *z*-motion error of *σ*_*z*_ = 42 nm. In (b), the dynamic error is varied by changing the camera exposure time (25, 250, and 2500 μs) while maintaining the signal-to-noise ratio across measurements by adjusting the illumination intensity. The static error is fixed at *σ*_S_ = 3 nm, and the corresponding dynamic errors are *σ*_D_ = 5, 8, and 12 nm, respectively. In (c), the static error is varied by tuning the illumination intensity to achieve different signal-to-noise ratios (SNRs ranging from 120 to 13, as indicated in the legend), while keeping the exposure time fixed at 25 μs (*σ*_D_ = 5 nm), and the corresponding static errors are *σ*_S_ = 3, 5, 10, and 20 nm, respectively. In all panels, the solid lines represent model fits using the hard-sphere potential, incorporating the relevant experimental errors. In panel (a), the best-fit particle radius is 261 nm, while in panels (b) and (c), it is 254 nm.

The *z*-motion error, *σ*_*z*_, is often difficult to determine directly in experiments. However, insights can be gained by analysing the *y*-motion (lateral, perpendicular to the long axis of the line trap), which is experimentally accessible. The *z*-motion is typically slightly larger than the *y*-motion, with the exact relationship depending on the particle size. It has been reported in ref. 35 that, for particles around *R* ∼ 250 nm, the trap stiffness in the *y*-direction is approximately 5 to 6 times higher than that in the *z*-direction. Based on this, we estimate 
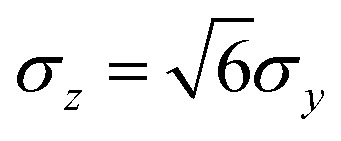
 for the measurements shown in Fig. 2(b) and (c). This estimation is consistent with values reported in previous studies using a similar AOM-based setup with slightly larger particles.^21^ For experiments conducted under fixed optical trapping conditions, *σ*_*z*_ is expected to remain constant, as it reflects the intrinsic properties of the trap. The scenario where the effects of the *z*-motion error on the measured potential are minimised depends on the minimisation of spherical aberration and a careful balance between trapping laser power and push. It is worth noticing that when two particles are being strongly pushed against each other by the lateral confinement of the line trap, the trend for particles to stack on top of each other is enhanced. This “over-pushing” in turn leads to an elevated *σ*_*z*_. In the present study, the effect of over-pushing is not included in *σ*_*z*_ as the particles are only gently pushed.

We also briefly discuss how gravity affects the axial motion of trapped particles. For a medium of density *ρ*_m_, a particle of density *ρ*_p_ and volume *V* has a gravitational length *l*_g_ = *k*_B_*T*/(*ρ*_p_ − *ρ*_m_)*V*g which measures the vertical distance over which gravitational potential energy changes by an amount comparable to the thermal energy *k*_B_*T*. For displacements Δ*z* ≪ *l*_g_, upward and downward thermal motions are equally likely. In this regime, the *z*-motion is dominated by optical trap confinement, and gravitational effects are negligible. The gravitational lengths of our *R* = 250 nm and *R* = 355 nm particles are *l*_g_ ≈ 121 μm and *l*_g_ ≈ 42.3 μm, respectively. These values are orders of magnitude larger than the measured nanoscale *z*-motion error *σ*_*z*_ in our experiments. In contrast, for much larger particles (*R* > 2 μm), and in particular for a larger buoyancy mismatch *ρ*_p_ − *ρ*_m_, *l*_g_ becomes comparable to *σ*_*z*_, and gravity can significantly suppress thermal *z*-direction fluctuations, thereby reducing the axial positional uncertainty. However, before reaching this limit, and even for high numerical aperture objectives, scattering forces become important, exerting radiation pressure and pushing particles along the optical beam propagation direction.^36^ Beads larger than *R* ∼ 2 μm are rare in precision tweezer work. They rapidly sediment and experience dominant scattering forces. Here, we consider only the common case where the gradient (tweezing) forces exceed the scattering forces and gravity can be neglected. However, if needed, such effects could easily be included in the analysis for specific cases where larger particles are studied.

Following the method reported in our previous work,^27^ the dynamic error *σ*_D_ is quantified by analysing the dependency of relative motion on a lag time. Specifically, all the frames from a video with a certain particle distance are selected. Then, the relative motion as a function of lag-time *τ* is calculated as the average change of distance with respect to the initially selected frames. Finally, the dynamic error is calculated from the interpolated *τ*-dependent relative motion (see the SI).

Static errors *σ*_S_ for different illumination settings are obtained experimentally by looking at the tracking variance of a pair of “in contact” particles adsorbed on the coverslip.^24^ Since the distance does not change any longer when they are adsorbed, the variance then reflects the tracking precision, or the static error.

## 3 Results

### Validation of the model

To validate our error model, we first establish a baseline condition corresponding to the best possible experimental settings in this set of measurements. These optimal parameters were determined through systematic tuning of the trapping depth, exposure time, and illumination intensity (see the SI). The trapping depth was adjusted to minimise spherical aberration, resulting in minimised axial fluctuations. As described in Section 2, the *z*-motion error is estimated from the lateral fluctuations. Specifically, we observe that the standard deviation in the *y*-direction is around *σ*_*y*_ = 17 nm at the optimised depth. Considering isotropic thermal motion and a harmonic potential, the axial localisation error is then given by 
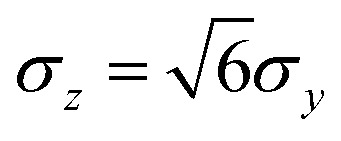
, leading to *σ*_*z*_ = 42 nm. A short exposure time of 25 μs was used to minimise the time-averaging effect, yielding a dynamic error of *σ*_D_ = 5 nm. Finally, sufficient illumination was applied to achieve a high signal-to-noise ratio in the recorded images, resulting in a static tracking error of *σ*_S_ = 3 nm. These baseline values define the reference for all subsequent measurements used to validate the model. The designed experiments systematically increase one source of error at a time—by deliberately defocusing the trap to increase *σ*_*z*_, extending the exposure time to increase *σ*_D_, or reducing illumination to increase *σ*_S_—while keeping the other parameters fixed. This controlled approach allows us to disentangle the contributions of each individual error source to the measured interaction potential and directly compare them with the predictions of the theoretical model.

Fig. 2 shows the measured potential for different experimental settings, designed to disentangle the effects of different errors on the extracted pair potential of polystyrene particles with a nominal radius of 250 nm. The particles were dispersed in 5 mM KCl (Debye length *λ*_D_ ∼ 4.3 nm) to minimise electrostatic interactions while still balancing the van der Waals interaction to prevent instability. Since the range of the double-layer interaction is smaller than the resolution of our setup, we model the interaction using an ideal hard-sphere potential for simplicity. In practice, we leave the particle radius as a fitting parameter to account for possible size variations. As shown in Fig. 2, the fitted radii in all three panels are slightly larger than the nominal value, which could be due to screening effects or simply arise from polydispersity.

The effect of the *z*-motion error was examined by changing the trapping depth within the sample, as shown in Fig. 2(a). Since spherical aberration varies with the focal plane position,^33,34^ adjusting the trapping depth modifies the extent of the out-of-plane motion. Experiments were performed with different trapping depths, resulting in different *z*-motion. The value of *σ*_*z*_ is fitted to be 76 nm, 46 nm, 42 nm (optimal position at depth *z* = 2.2 μm), 50 nm, and 58 nm, respectively, as moving the trapping depth from very close to the coverslip surface to 4.4 μm into the sample. All measurements of this group were conducted with a fixed exposure time of 25 μs (dynamic error *σ*_D_ = 5 nm) and with sufficient illumination (static error *σ*_S_ = 3 nm). This set of experiments serves two purposes: first, to quantify the effect of the *z*-motion error on the extracted potential; and second, to determine the optimal experimental conditions that minimise this error. The results indicate that when spherical aberration is minimised by carefully choosing the trapping depth, *z*-motion fluctuations are significantly reduced, allowing a more accurate potential measurement to be obtained. This is the case for the potentials plotted with green and orange colours in Fig. 2(a).

The measurements in Fig. 2(b) and (c) were performed at the optimised depth, where the *z*-motion error was estimated to be approximately *σ*_*z*_ = 42 nm, as detailed in the beginning of this section. Fig. 2(b) explores the influence of the dynamic error on the measured potential. The dynamic error arises from time-averaging due to the finite exposure time, which systematically shifts the extracted pair potential. To quantify this effect, we recorded measurements with exposure times of 25 μs, 250 μs, and 2500 μs. The SNR of the recorded images was maintained by adjusting illumination intensity accordingly. The corresponding dynamic errors were *σ*_D_ = 5, 8, and 12 nm, while the static error was fixed at *σ*_S_ = 3 nm. The results indicate that increasing exposure time does not significantly alter the overall shape of the potential but causes a slight shift in the apparent particle size. This shift occurs because longer exposure times bias the recorded particle separation towards larger distances (in the case of repulsive interaction) due to the averaging effect over fluctuating positions. For attractive potentials, the dynamic error has a different influence: it smears the attraction potential, making it appear shallower, as shown in Fig. 1(b).

Fig. 2(c) examines the role of the static error, which is associated with localisation uncertainty due to finite tracking precision. This error depends primarily on the SNR of the recorded images, which was systematically varied by tuning illumination intensity. The experiments were conducted with a fixed exposure time of 25 μs to ensure that the dynamic error remained constant (*σ*_D_ = 5 nm). Four different illumination intensities were used, corresponding to SNRs of 120, 40, 20 and 13 (static errors of *σ*_S_ = 3, 5, 10, and 20 nm). The results confirm that a higher static error leads to a broader and softer apparent interaction potential, as expected. The experimental data in Fig. 2(b) and (c) are well-matched with ideal hard-sphere interaction models that incorporate the corresponding error contributions.

### Correction of the errors

With the validation of the error model, we now turn to establishing a correction framework that allows for the extraction of accurate interaction potentials from experimental data affected by these errors. The approach to correction depends on whether the functional form of the potential is known or not.

When the form of the potential is unknown, the problem becomes more general and falls into the category of inverse problems. Extracting the true potential in this case requires regularisation techniques to prevent overfitting and ensure stability in the solution.^37,38^ More advanced methods, such as machine learning approaches including neural networks, have also been proposed to tackle inverse problems in experimental physics.^39,40^ However, the solution of inverse problems is beyond the scope of this work. Here, we focus on demonstrating an effective correction method for the case where the potential form is known and leave the general inverse problem for future studies. Nonetheless, we would like to emphasize that a systematic variation of the contributions from different error sources, as demonstrated in this work, could provide key input data for performing a stable inversion.

In cases where the form of the potential is known, the correction can be performed by applying the error models to the true potential and tuning the adjustable parameters so that the blurred true potential matches the measured data. This allows us to reconstruct the true interaction potential by systematically removing the distortions. We demonstrate this approach with the well-known case of depletion attraction.

Depletion interactions arise in colloidal suspensions due to the exclusion of smaller particles from the space between larger ones, leading to an effective attraction. The Asakura-Oosawa (AO) model^41^ describes this interaction with a well-defined potential form, making it an ideal case study for validating our error correction framework. According to the AO model, the depletion potential *U*_AO_(*h*) between two colloidal particles of radius *R* in a suspension of non-adsorbing depletant of diameter *d*_m_, in the case of *d*_m_ ≪ *R*, is given by:5
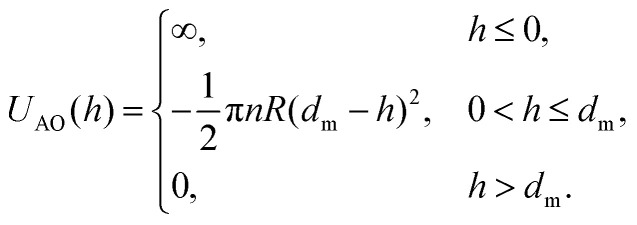
Where *n* represents the number density of the depletant, and *h* = *r* − *R* is the surface–surface distance of the large particles. The AO model considers the depletant (small) particles to behave like an ideal gas, and therefore, it is most accurate when applied to low to moderate depletant densities.

To validate our correction method, we measured the depletion potential between two trapped polystyrene particles of radius *R* = 355 nm in the presence of a non-adsorbing micelle solution. The depletion interaction measurements were performed under optimised conditions to minimise experimental errors. The measurements were conducted in a PBS 1X buffer to maintain a stable pH of 7.4. In this buffer, the Debye length is approximately *λ*_D_ ≈ 0.7 nm.^42^ As the depletant, we used Pluronic F108 copolymer micelles to induce short-range attractive interactions, performing measurements at two different F108 concentrations, 1 mM and 2 mM. As reported in ref. 43, the critical micelle concentration (CMC) of F108 is temperature-dependent. Our experiments were conducted at *T* = 35 °C, where the CMC is approximately 0.15 mM. The surfactant concentration was chosen sufficiently low to ensure that liquid structuring effects in the micellar solution remain negligible. Concerning the latter, the contact potential deepens to a leading order as (1 + 0.2*ϕ*).^44^ Since we remain within *ϕ* ≤ 0.12, this contribution remains negligibly small—see also the SI in our ref. 27

**Fig. 3:**
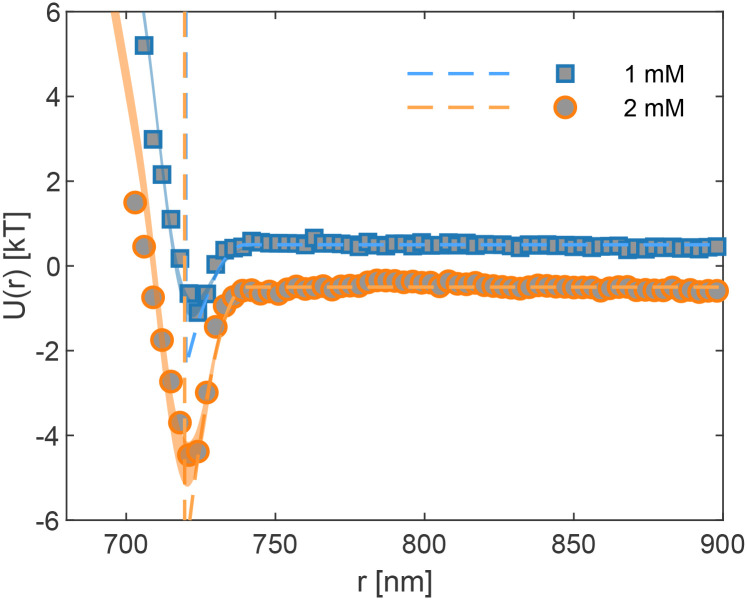
Measured depletion interaction potential *U*(*r*) between polystyrene colloids (*R* ≃ 355 nm, nominal size) in Pluronic F108 solutions (1 mM and 2 mM) prepared in PBS 1X at *T* = 35 °C. Symbols represent experimental data. Shaded areas show the numerically modelled potentials using the Asakura Oosawa (AO) framework, incorporating experimental errors: *σ*_*z*_ = 42 nm, *σ*_D_ = 5 nm, and *σ*_S_ = 3 nm. The AO model parameters include a polystyrene particle radius set to *R* = 360 nm for best agreement with experiments (slightly larger than the nominal mean 355 nm), a micelle diameter of *d*_m_ = 20 nm determined by DLS, and a micelle aggregation number chosen in the range of 40 ± 4. Dashed lines show the unblurred AO model for comparison, plotting with an aggregation number of 40. This value results in micelle number densities *n* of 12.8 × 10^3^ μm^−3^ for 1 mM and 27.8 × 10^3^ μm^−3^ for 2 mM.

Taking the AO model, eqn (5), and numerically accounting for the different sources of experimental uncertainty according to eqn (1)–(4), we obtain a version of the AO model blurred by experimental errors. This blurred model is directly compared to the experimentally measured interaction potentials shown in Fig. 3, where the shaded areas represent the range of plausible interaction potentials. In this analysis, the colloidal particle radius *R*, the micelle diameter *d*_m_, and the micelle number density *n* are the relevant parameters. The particle radius is set to *R* = 360 nm to achieve the best agreement, slightly larger than the nominal value of 355 nm, a difference which may arise from polydispersity. The micelle diameter is fixed at *d*_m_ = 20 nm, as determined independently by dynamic light scattering (DLS, NanoLab 3D, LS Instruments, Switzerland). The micelle number density *n* is derived from the aggregation number, chosen to be 40 ± 4, following the literature††For F108 micelles of diameter 14, 22, and 28 nm, the aggregation numbers are predicted to be 35, 43, and 61, respectively..^43,45^ An aggregation number of 40 yields *n* values of 12.8 × 10^3^ μm^−3^ (volume fraction *ϕ* = 5.4%) for 1 mM and 27.8 × 10^3^ μm^−3^ (*ϕ* = 11.7%) for 2 mM. As shown in Fig. 3, the experiments fall very well within the prediction by eqn (5).

The dashed lines in Fig. 3 represent the unblurred AO potential, plotted using the same parameters as the solid lines. It is evident that, without accounting for experimental errors, a quantitative interpretation of the measured potential is not possible. The discrepancy between the unblurred AO model and the experimental data highlights the necessity of incorporating experimental uncertainties into the theoretical framework. By applying the error correction model, we effectively reconstruct the true interaction potential, demonstrating the robustness of our approach.

## 4 Discussion and conclusions

In this work, we have systematically studied experimental errors in the measurement of pair interaction potentials using line optical tweezers (LOT). Through controlled experiments, we validated a model that accounts for three primary sources of errors: *z*-motion error, dynamic error, and static error. Each of these errors contributes to distorting, shifting and broadening the extracted potential in distinct ways. Understanding these effects allows for a systematic correction approach, improving the reliability of experimentally extracted potentials.

With this validated error model, we established a correction framework that enables the accurate extraction of true interaction potentials from experimental data. We demonstrated the effectiveness of this approach using a depletion interaction showcase, where the experimentally measured potential, affected by systematic errors, was corrected to recover the underlying Asakura-Oosawa (AO) depletion potential. By systematically removing the effects of experimental distortions, we showed that precise reconstruction of interaction potentials is possible.

Overall, this study provides a practical guideline for performing precise potential measurements using optical tweezers and represents a significant step toward achieving absolute potential measurements in soft matter systems. By systematically identifying and correcting key sources of errors, our framework enhances the reliability of experimental data, enabling more precise comparisons with theoretical models and simulations. Future work may extend this methodology to explore data-driven, model-free approaches for extracting interaction potentials from experimental measurements.

## Author contributions

CZ was responsible for conceptualisation and methodology and provided guidance to JMD throughout the project. JMD conducted the majority of the experiments. CZ and JMD performed data analysis. AM and LFP carried out the numerical calculations. FS provided the overall supervision of the project. JMD and CZ led the writing of the original draft. All authors contributed to writing, review and editing and participated in data analysis.

## Conflicts of interest

There are no conflicts to declare.

## Supplementary Material

SM-021-D5SM00551E-s001

## Data Availability

All experimental and numerical data generated in this study have been deposited in the repository Zenodo under the accession code https://doi.org/10.5281/zenodo.15838908. All additional data sets generated during and or analysed during the current study are available from the corresponding author upon request. Supplementary information is available. See DOI: https://doi.org/10.1039/d5sm00551e.
