# Supplementary material for: Quantifying experimental errors in measuring colloidal interaction potentials with optical tweezers
Source: Soft Matter. 2025 Sep 1;21(39):7622–30. doi: 10.1039/d5sm00551e (PMC12439875; doi:10.1039/d5sm00551e)
Supplement: SM-021-D5SM00551E-s001 [file SM-021-D5SM00551E-s001.pdf]

Supplementary Information (SI) for Soft Matter.
This journal is © The Royal Society of Chemistry 2025

Supplementary Material

José Muñetón-Díaz[1], Augustin Muster[1], Luis S. Froufe-Pérez [1], Frank Scheffold [1‡], Chi Zhang [1⋆]

[1]Department of Physics, University of Fribourg, 1700 Fribourg, Switzerland

‡frank.scheffold@unifr.ch

⋆chi.zhang2@unifr.ch

Inter-particle potential analysis

The data analysis to reveal the intrinsic potential follows our previous work [1]. Briefly, the measured pair potential $U_{\text{raw}}(r)$ converted from the distribution of the inter-particle distance contains three components:

$$U_{\text{raw}}(r) = U_{\text{OT}}(r) + U_{\text{OB}}(r) + U(r), \tag{S1}$$

where $U(r)$ represents the intrinsic inter-particle interaction, $U_{\text{OT}}(r)$ the harmonic potential of the LOT, and $U_{\text{OB}}(r)$ the optical binding (OB) potential. Careful removal of $U_{\text{OT}}(r)$ and $U_{\text{OB}}(r)$ allows appropriate extraction of $U(r)$.

The harmonic trapping potential is modelled with the formula $U_{\text{OT}}(r) = \frac{1}{2}kr^2$, where k represents the line trap stiffness and can be easily extracted from single particle measurements.

For static LOT (SLM based), we have shown in our previous work [1] that it can be well expressed as the following empirical equation.

$$U_{\text{OB}} = A\cos[(2\pi r/\lambda + \phi)]/(r/2R - \alpha), \tag{S2}$$

where A represents the oscillation amplitude, λ is the wavelength of the trapping laser, ϕ corresponds to the phase, R is the particle radius, and α is an empirical parameter of order one (we use $\alpha = 0.8$, as found to be a good match in [1]).

For the time-shared LOT (AOM based), it is found that the OB is quite different. Instead of oscillating and reaching to far range, it vanishes relatively quickly as the inter-particle distance increases. We will discuss the difference of OBs generated from different LOTs in the next section. To model the OB, we found that adding a decaying term captures the shape of the potential well.

$$U_{\text{OB}} = A\cos[(\pi r/\lambda + \phi)]/(r/2R - \alpha)\exp\left(-(r/\sigma_{OT})^2\right), \tag{S3}$$

We introduced the exponential decay term $\exp\left(-(r/\sigma_{OT})^2\right)$ in the optical binding (OB) potential for the time-shared line optical tweezers (LOT) to empirically capture the rapid decay of the interaction observed in experiments. We found that $\sigma_{OT} \approx 500$ nm accurately describes the shape of the OB in our measurements. Notice that the first term in this expression gives a larger oscillation length compared to equation S2, which describes the static LOT case. The inclusion of this decay term is primarily for empirical convenience, as it provides an excellent description of both the experimental measurements and the DDA calculations, as shown in Figure S2. Ultimately, the force difference for both OT illumination schemes arises from the fact that the electromagnetic field distribution around the particles is different at any given time even if, on average, the intensity of the trap is the same. Hence, the time average optical force depends on the illumination scheme even for equal averaged intensity.

To remove $U_{\text{OT}}(r)$ and $U_{\text{OB}}(r)$, the measured two particle raw potential is fitted using the equations listed above, at the far range where the intrinsic interaction effectively vanishes. Figure S1 shows how the intrinsic potential is extracted.

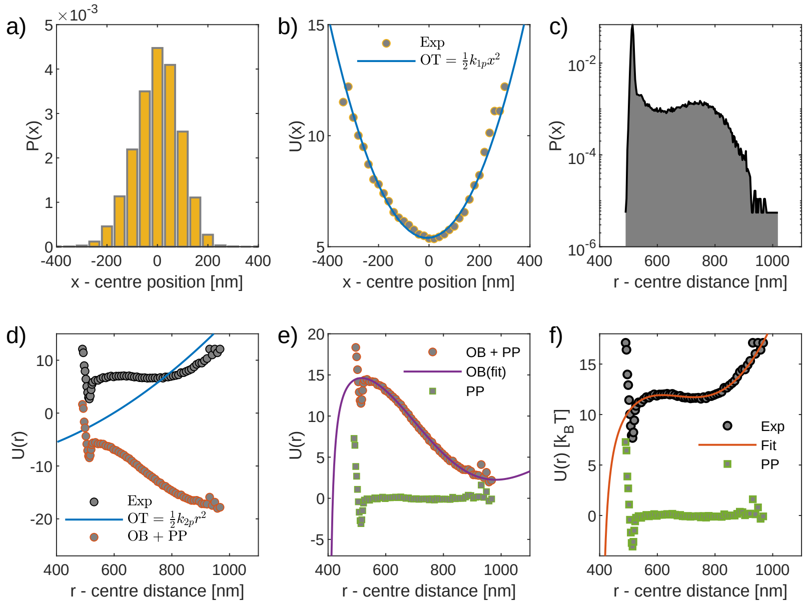

Figure S1: Extraction of the intrinsic potential. The measured distribution of position for a single particle (a) and the inter-particle distance distribution for two particles (c) can be converted to potentials via the Boltzmann relation. (b) The single-particle potential is fitted to obtain the trap stiffness k_{1p}. (d) For two particles, the effective stiffness is $k_{2p} = \frac{1}{2}k_{1p}$, which can be subtracted, leaving the intrinsic pair potential (PP) and the optical binding (OB) potential. (e) The OB potential is removed by fitting at large distances where the intrinsic interaction vanishes, using the empirical equations S2 or S3. (f) In practical cases where the interaction is measured over a sufficient range, $U_{\mathrm{OT}}(r)$ and $U_{\mathrm{OB}}(r)$ can be removed in a single step by combining them and fitting the raw potential at large distances.

Characterisation of optical binding potential

Optical binding (OB) is a phenomenon where long-range forces arise between microscopic dielectric objects in an intense optical field, leading to stable or oscillatory bound states due to the scattered electromagnetic fields [2]. Since OB effects fundamentally arise from light scattering, it follows that the specific manner in which a line trap is created will influence the resulting OB potential. Since time-shared and holographic-static line traps differ in their temporal, scattered optical field distributions, they also show distinct optical binding interactions.

To obtain the OB potential, we study polystyrene particles with a radius of 250 nm dispersed in 5 mM of KCl solution, where the Debye length is about 4.3 nm. The screening is strong enough to reduce the double-layer electrostatic interaction to a very short range, while still maintaining sufficient short-range repulsion to prevent instability induced by Van der Waals interaction. Hence, in this case, the intrinsic potential is effectively nulled in the non-contacting range. Therefore, the measured raw potential (as in equation S1) consists only of $U_{\mathrm{OT}}(r)$ and $U_{\mathrm{OB}}(r)$. The optical trapping potential can be obtained via single-particle measurements. With the experimental distribution of positions of a single particle in the OT (see fig. S1a), the single-particle potential $U^{1p}_{\mathrm{OT}}(r)$ is extracted via the Boltzmann relation and fitted to a harmonic potential $U^{1p}_{\mathrm{OT}}(r) = \frac{1}{2}k_{1p}r^2$ (see fig. S1b). It can be shown that the 2-particle optical trapping potential is a harmonic potential $U_{\mathrm{OT}}(r) = \frac{1}{2}k_{2p}r^2$ with a stiffness $k_{2p} = \frac{1}{2}k_{1p}$ [1].

[1] The total energy of two particle in the trap can be written as $U_{OT}(x_1, x_2) = \frac{1}{2}k_{1p}x_1^2 + \frac{1}{2}k_{1p}x_1^2$. Replacing x_1 and x_2 with the centre of mass M and distance r ($x_1 = M + r/2$ and $x_2 = M - r/2$), it is easy to derive that $U_{OT}(M, r) = k_{1p}M^2 + \frac{1}{4}k_{1p}r^2$ This

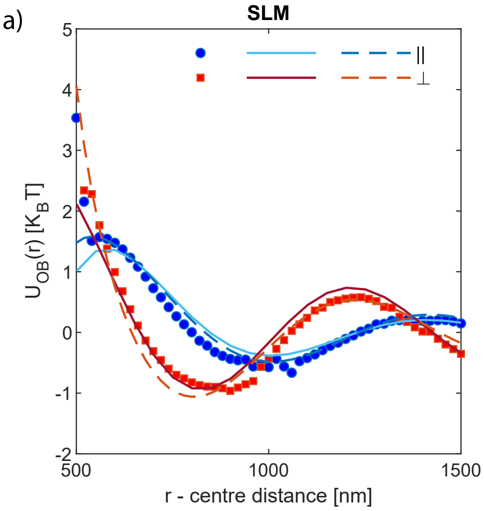

Figure S2: Optical binding potentials of two $R = 250$nm polystyrene particles: measurements (symbols), DDA calculations (solid lines) and description by empirical equations S2 and S3 (dashed lines). Panel (a) shows the OB potentials extracted from a static LOT generated with SLM, whereas the potentials in (b) are from a time-shared line generated with the AOM setup. All the potentials are rescaled so that they correspond to the case that is measured in a trap with laser power about 30 mW/μm.

Analogously, the total interaction potential can be extracted form the measured centre-to-centre distance distributions (as shown in fig. S1c) by using the Boltzmann relation again. Since the optical trapping is known independently, the total interaction potential $U_{\rm OB}(r) + U_{\rm PP}(r)$ is determined by subtraction of $U_{\rm OT}(r)$ to the total potential (see fig. S1d). The intrinsic pair potential $U_{\rm PP}(r)$ is determined by subtracting the optical binding potential found by fitting the total interaction potential to the empirical equations S2 or S3, as adequate, at large distances only, where the intrinsic pair interaction is forced to be effectively null (see fig. S1e). Nonetheless, in many practical cases the distances range is long enough to allow for the removal of $U_{\rm OB}(r)$ in a single step (see fig. S1f).

The measurements of OB with SLM are well described in our previous work [1], with two truncated short Gaussian traps. With the AOM, OB was measured with a relatively shallow long Gaussian trap (about 4 μm in length). With both setups, OB were studied with the polarisation of the trapping laser in both parallel and perpendicular directions to the long axis of the line.

Fig. S2 shows OB potentials in the static and time-shared line setups, for experimental measurements (symbols), discrete dipole approximation (DDA) calculation (solid lines), and empirical equations S2 and S3 (dashed lines). We investigate the differences in optical binding between these two experimental setups. The static LOT generates an optical binding interaction characterised by oscillations that extend over long distances, with a slow decay following an approximate $1/r$ scaling. The oscillations can be described well with a period with a length of λ. In contrast, the time-shared LOT produces an optical binding potential with minimal oscillations, which decays more rapidly as the inter-particle distance increases. If we try to describe the OB potential as a product of an oscillating part and a decaying part, as shown in equation S3, the oscillation period aligns more with a length of 2λ. It is likely that one can find a better empirical equation rather than S3. However, it is beyond the scope of this work. Here, we want to make the form of the formula similar to equation S2 for comparison and for simplicity.

decomposition shows that the centre of mass M experience a harmonic potential with stiffness of $2k_{1p}$, while the relative coordinates, or the distance, experiences a harmonic potential with stiffness of $\frac{1}{2}k_{1p}$.

Optimisation of experimental conditions

Optimal experimental conditions are achieved by minimizing the three errors. Dynamic error is intuitively optimised by reducing the exposure time. On the other hand, excessively short exposure times reduce photon statistics - given the illumination power is finite - which increases static error. In some cases, the influence of slightly elevated dynamic error is not so pronounced, as shown in Figure 2 of the main text. Conversely, increasing exposure time can significantly reduce static error when the photon budget is already low. In this case, it is generally preferable to choose an exposure time that first prioritises minimizing static error. For instance, in Figure 2(c) of the main text, the average intensities of the images with signal-to-noise ratios SNR (ratio between the signal amplitude ($I_{max} - I_{min}$) and the background noise level, SD($I_{background}$), where SD is the standard deviation) of 13, 20, 40, and 120 are considered. One can see that at lower photon budgets, a small increase in intensity can significantly reduce static error. Of course, when contrast is good and photon statistics are sufficient, as in the case of SNR above 20, one should record images with the lowest possible exposure time.

The optimisation of z-motion error depends on a few factors. First, there is longitudinal spherical aberration (LSA), which can hinder trap stiffness. LSA originates from the quality of the laser beam, the choice of lenses and objectives, and the alignment of the optical setup. Spherical aberration causes rays from different parts of the laser beam to converge at different positions, preventing the beam from being tightly focused. Since spherical aberration varies with the focal plane position, adjusting the trapping depth modifies the extent of out-of-plane motion. A first set of calibration measurements is made with the optical trap placed at different depths in the sample in order to choose the depth at which the contribution of LSA to the error σ_z is minimised. This is shown in Figure 2(a) in the main text.

The second factor affecting the z-motion error is the trapping (laser power per unit length)–pushing (length of the laser intensity profile–σ of the Gaussian trap) balance, which influences the stiffness k_z and trapping efficiency in the z direction. Increasing laser power enhances k_z, thereby reducing out-of-plane fluctuations. However, excessively high laser power can also increase unwanted optical binding effects. Additionally, high power often results in over-pushing, causing particles to remain near the centre of the line trap for extended periods, reducing the number of sampled positions and potentially biasing the interaction potential by limiting the available range of separations. Over-pushing also increases the tendency of particles to stack on top of each other or even swap positions, leading to elevated σ_z. Moreover, excessive overlap between particle images can interfere with the tracking algorithm, introducing additional errors.

To achieve a decent trapping efficiency without introducing over-pushing, one can use a shallow Gaussian profile with sufficient laser power. However, the configuration of the LOT requires experience and should be designed specifically for a targeted interaction.

Results of time-shared LOT

The results similar to Figure 2(b) and (c) in the main text, measured with the time-shared LOT (AOM based), are shown in Figure S3. Although the AOM and SLM create LOTs through distinct mechanisms and generate different OB potentials, the extracted intrinsic potentials are very similar. The values of σ_z, σ_D, and σ_S are also comparable under similar experimental settings.

Optical binding with discrete dipole approximation (DDA)

The computation of the optical binding between two particles in a line trap generated by an AOM is done using the Discrete Dipole Approximation (DDA) method implemented in the CoupledElectricMagneticDipoles.jl julia package [3]. We model each of the two PS particles (radius a = 260 nm, refractive index n = 1.59) immersed in water (refractive index n_h = 1.33) by discretizing it into N = 720 electric dipoles with radiative-corrected [4] Clausius-Mossotti polarizability (a convergence study of the DDA method is available in the next section).

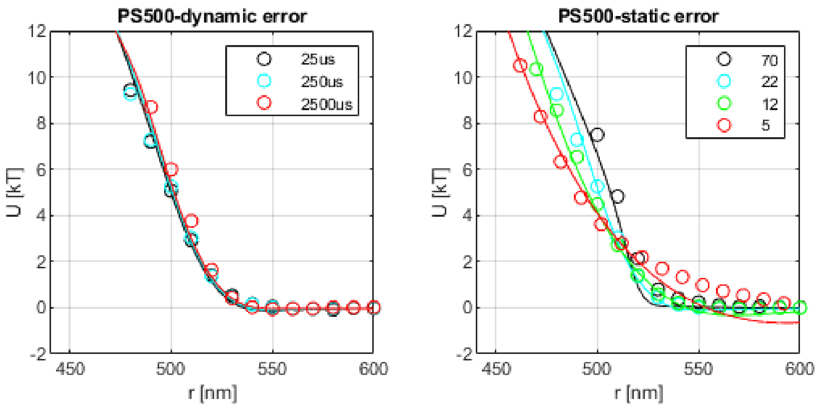

Figure S3: Influence of dynamic error (a) and static error (b) on experimentally extracted (AOM, time-shared LOT) pair potentials on polystyrene particles with radius of 250 nm. The measurements for (a) and (b) were performed at the same depth. Data shown in (a) were measured with different exposure time of 25μs, 250μs and 2500μs, thus changing the dynamic error. The illumination intensity was tuned to keep the photon statistics of the recorded images similar. Data shown in (b) were measured with the same exposure time of 250μs, while the intensity was tuned to get different SNR (70 to 5 as listed in the legend), thus changing the static error. The static error in (a) is $\sigma_S = 10$ nm with dynamic error of $\sigma_D = 5$, 8 and 12 nm, respectively. The dynamic error in (b) is $\sigma_D = 8$ nm with static error of $\sigma_S = 3$, 10, 20 and 40 nm, respectively. The z-motion is the same for all measurements in (a) and (b), as $\sigma_z = 47$ nm. Solid lines in (a) and (b) are interactions modelled using hard sphere potential with corresponding experimental errors.

The optical binding force F_{OB}^{A} acting on particle A in an AOM generated line trap is computed as the intensity-weighted average force over all focal points r_f:

$$F_{OB}^{A}(r) = \int_{r_{min}}^{r_{max}} dr_f \, I(r_f) \, F_{OB}^{A}(r, r_f) \, / \int_{r_{min}}^{r_{max}} dr_f \, I(r_f) \tag{S4}$$

Where $I(r_f)$ is the measured intensity profile at the different focal points r_f along the line trap, as shown in figure S4. In this work we discretise the integration interval $r_f \in [-2.25 \; \mu m, 2.25 \; \mu m]$ using N_f =41 points. The Gaussian beam's wavelength in water is $\lambda = 800$ nm, and its focal spot has a FWHM = 274 nm. $F_{OB}^{A}(r, r_f)$ is measured by computing the difference between the total optical force on particle A in the presence and in absence of particle B (denoted w/B and w/o B resp.): $F_{OB}^{A}(r, r_f) = F_{O}^{A,\text{ w/B}}(r, r_f) - F_{O}^{A,\text{ w/o B}}(r, r_f)$, where both forces are computed using the `Forces` module of CoupledElectricMagneticDipoles.jl. In order to get the optical binding potential U_{OB} , given by the relation $F_{OB}(r) = -\partial U_{OB}(r)/\partial r$, F_{OB} is computed in an interval of distances $r = [2a, 10 \; \mu m + 2a]$ and integrated using a Simpson integration scheme. Calculations are repeated for both parallel and perpendicular polarisations.

Convergence of the DDA Method

In order to show the convergence of the DDA method, we compute F_{OB} for several levels of discretisation (from 117 to 1256 dipoles per particle) and a Gaussian beam placed at the centre between the two particles. Figure S5 shows the obtained results, showing that the use of $N = 720$ dipoles per particle keeps the absolute error within 2 percent of the maximum absolute force for the finest discretisation level.

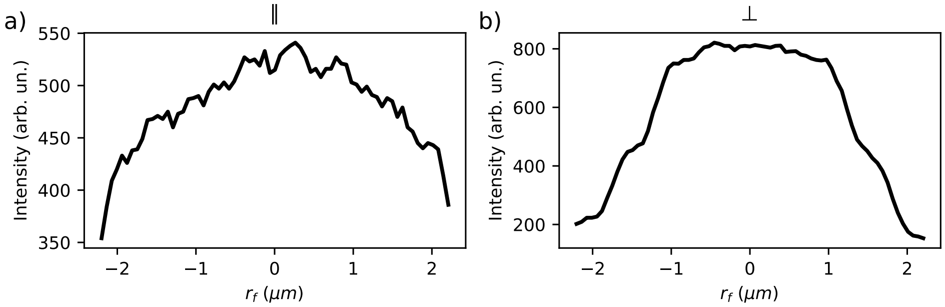

Figure S4: Intensity profile of the line trap $I(r_f)$ used to perform the numerical computations for a) parallel, and b) perpendicular polarisation.

Dynamic Error

In this section, we discuss the dynamic error introduced by finite camera exposure time in potential measurements using optical tweezers (OT). For simplicity, hydrodynamic coupling between particle pairs is neglected. The motion of the particles is driven by thermal fluctuations and interparticle interactions. The thermal component is uncorrelated and well-described by Brownian motion, while the interaction component is correlated and introduces fluctuation-induced biases in the measured particle separation.

At the start of an exposure, assume the particle pair is at a distance r. During the exposure time, thermal motion causes their separation to fluctuate continuously. The recorded distance is the average of this fluctuating trajectory and differs from the starting distance by a deviation a, such that the recorded distance is $r + a$. The probability of observing a given averaged deviation a from position r is described by the distribution $Q_{\mathrm{D}}(r, a)$. We discuss here how to derive this $Q_{\mathrm{D}}(r, a)$.

In the following analysis, we focus on the position of a single particle in the trap. Since hydrodynamic coupling is neglected, the analysis for the interparticle distance is analogous to that of a single particle.

Free diffusion

We consider a particle undergoing free overdamped Brownian motion in one dimension. The motion is described by the Langevin equation:

$$\frac{dx(t)}{dt} = \sqrt{2D}\,\eta(t), \tag{S5}$$

where D is the diffusion coefficient, and $\eta(t)$ is a Gaussian white noise process with zero mean and unit variance, representing thermal fluctuations. We assume the particle starts at the origin, $x(0) = 0$.

Integrating this stochastic differential equation yields the position of the particle as a function of time:

$$x(t) = \sqrt{2D}\,W(t), \tag{S6}$$

where $W(t)$ is a standard Wiener process. This process characterises Brownian motion with diffusion coefficient D, and its statistical properties are:

- $x(0) = 0$
- $\langle x(t) \rangle = 0$
- $\langle x(t_1)x(t_2) \rangle = 2D \cdot \min(t_1, t_2)$

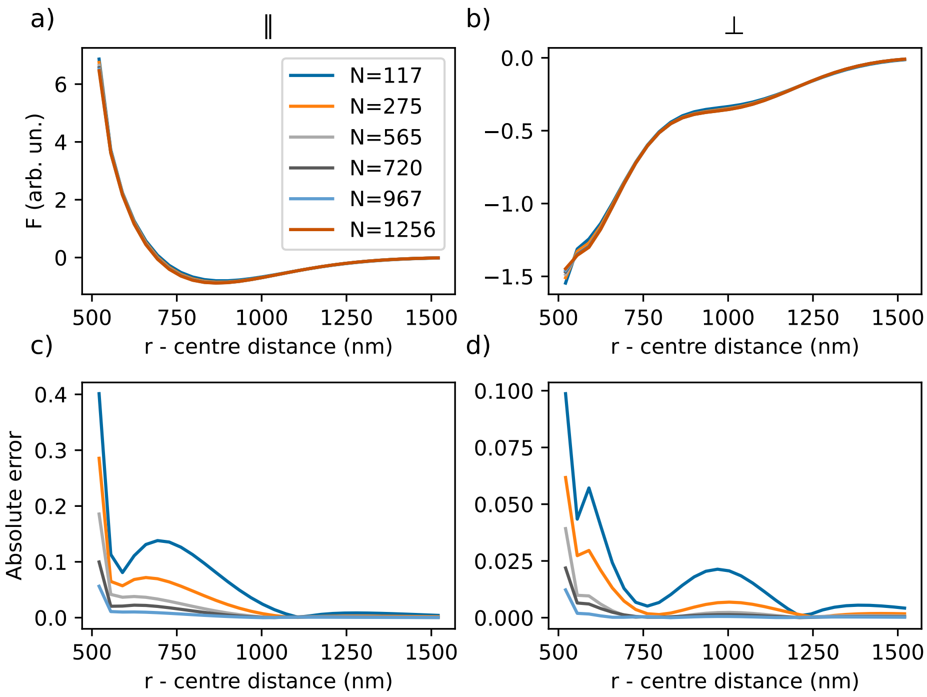

Figure S5: Convergence of the DDA method. a)-b): The optical binding force F_{OB} for several levels of discretisation, for both parallel (panel a)) and perpendicular polarisations (panel b)). c)-d): Absolute error of F_{OB} with respect to the finest ($N = 1256$) discretisation level.

In particular, this implies that $x(t)$ is a Gaussian process, which allows us to analytically compute the distribution of time-averaged displacements during the exposure time.

The recorded position in the image reflects the average of this fluctuating trajectory over the exposure time t_{exp}. We define the time-averaged deviation a as:

$$a = \frac{1}{t_{\text{exp}}} \int_0^{t_{\text{exp}}} x(t)\, dt \tag{S7}$$

Therefore, a also follows a Gaussian distribution. We now compute the variance of a, denoted σ_D^2, using the definition:

$$\sigma_D^2 = \langle a^2 \rangle = \left\langle \left(\frac{1}{t_{\text{exp}}} \int_0^{t_{\text{exp}}} x(t)\, dt \right)^2 \right\rangle \tag{S8}$$

Substituting in the expression for a, we expand:

$$\sigma_D^2 = \frac{1}{t_{\text{exp}}^2} \int_0^{t_{\text{exp}}} \int_0^{t_{\text{exp}}} \langle x(t_1)x(t_2) \rangle\, dt_1\, dt_2 \tag{S9}$$

Using the autocorrelation property of the Wiener process [5]:

$$\langle x(t_1)x(t_2) \rangle = 2D \cdot \min(t_1, t_2) \tag{S10}$$

So we have:

$$\sigma_D^2 = \frac{2D}{t_{\text{exp}}^2} \int_0^{t_{\text{exp}}} \int_0^{t_{\text{exp}}} \min(t_1, t_2)\, dt_1\, dt_2. \tag{S11}$$

This double integral can be evaluated by splitting the domain into two symmetric halves, using the identity:

$$\int_0^{t_{\text{exp}}} \int_0^{t_{\text{exp}}} \min(t_1, t_2)\, dt_1\, dt_2 = 2 \int_0^{t_{\text{exp}}} dt_2 \int_0^{t_2} t_1\, dt_1 \tag{S12}$$

Therefore,

$$\sigma_D^2 = \frac{2D}{t_{\text{exp}}^2} \cdot \frac{1}{3} t_{\text{exp}}^3 = \frac{2Dt_{\text{exp}}}{3} \tag{S13}$$

This result shows that the average deviation a over the exposure interval follows a Gaussian distribution with zero mean and variance $\sigma_D^2 = \frac{2}{3} D t_{\text{exp}}$, which quantifies the dynamic blurring effect due to finite exposure time. Hence, the distribution of the deviation a is given by

$$Q_D(a) = \frac{1}{\sqrt{2\pi\sigma_D^2}} \exp\left(-\frac{a^2}{2\sigma_D^2}\right) \qquad \text{with } \sigma_D^2 = \frac{2Dt_{\text{exp}}}{3}. \tag{S14}$$

This distribution is independent of r, consistent with the assumption of free diffusion without external forces or potentials.

Diffusion on a shallow energy landscape

In the presence of interparticle interactions, particle motion occurs on a potential energy landscape rather than in free space. This landscape introduces a directional bias into the thermal fluctuations: displacements that lower the potential energy become more probable, while those that increase it are energetically suppressed. As a result, the distribution of time-averaged deviations during the exposure time becomes position-dependent and asymmetric. To accurately model the dynamic error under these conditions, we must incorporate the influence of the potential into the fluctuation statistics. This section presents a modified framework that accounts for such interaction-driven biases.

We consider a particle undergoing overdamped Brownian motion in one dimension under a potential energy landscape $U(r)$. The motion is governed by the Langevin equation:

$$\frac{dx(t)}{dt} = \mu F(r) + \sqrt{2D}\,\eta(t), \qquad F(r) = -\frac{dU}{dr}, \tag{S15}$$

where μ is the mobility, D is the diffusion coefficient, and $\eta(t)$ is a Gaussian white noise process with zero mean and unit variance. For simplicity, we assume the particle starts at the origin, $x(0) = 0$.

Assuming that the force $F(r)$ varies slowly over the particle's trajectory during the exposure time t_{exp}, we approximate it as constant. This allows us to linearise the dynamics and write the solution as:

$$x(t) = \mu F t + \sqrt{2D}\, W(t), \tag{S16}$$

where $W(t)$ is a standard Wiener process.

The average displacement over the exposure time is then given by equation S7. Substituting the expression for $x(t)$, we obtain:

$$a = \frac{1}{t_{\text{exp}}} \int_0^{t_{\text{exp}}} \left(\mu F t + \sqrt{2D} W(t)\right) dt = \mu F \frac{t_{\text{exp}}}{2} + \frac{1}{t_{\text{exp}}} \int_0^{t_{\text{exp}}} \sqrt{2D}\, W(t)\, dt. \tag{S17}$$

Since the integral of the Wiener process is Gaussian with zero mean and variance $\frac{2}{3}Dt_{\text{exp}}$, we conclude that the distribution of a is Gaussian with mean $\mu F \frac{t_{\text{exp}}}{2}$ and variance $\frac{2}{3}Dt_{\text{exp}}$. Therefore, the distribution of time-averaged displacement under a constant external force becomes:

$$G_D(r,a) = \frac{1}{\sqrt{2\pi\sigma_D^2}} \exp\left(-\frac{(a-\mu F(r)t_{\text{exp}}/2)^2}{2\sigma_D^2}\right), \qquad \text{with } \sigma_D^2 = \frac{2Dt_{\text{exp}}}{3}. \tag{S18}$$

This expression captures how both thermal fluctuations and deterministic drift contribute to the dynamic error in the presence of a potential gradient.

This derivation relies on the assumption that the force $F(r)$ is approximately constant over the trajectory of the particle during the exposure time. Physically, this corresponds to a slowly varying or shallow potential $U(r)$ in the region explored by the particle. While this approximation yields a closed-form result, it becomes invalid in regions where the potential changes significantly over the scale of thermal fluctuations. In such cases, the force is no longer constant, and the Langevin equation cannot be solved analytically without specifying the full functional form of $U(r)$. As a result, the probability distribution of time-averaged displacements becomes both position-dependent and non-Gaussian, making further analytical treatment difficult.

Coarse-grained modelling

To proceed without committing to a specific form of $U(r)$, we adopt a coarse-grained approach that captures the essential physics. The idea is that fluctuations that move the system to lower potential energy are favoured, while those that increase energy are suppressed. To account for this, we include an energetic weighting into the fluctuation model. The probability of a fluctuation from r to $r+a$ is given by:

$$Q_{\text{D}}(r,a) \propto \exp\left(-\frac{a^2}{2\sigma_D^2}\right) \exp\left(-U(r+a)\right), \tag{S19}$$

where $U(r+a)$, the potential at the recorded position, modulates the probability of such a fluctuation. When $U(r)$ is flat, Q_{D} reduces to a pure Gaussian. In regions of strong confinement or attraction, the Boltzmann term suppresses motion, effectively reducing the dynamic error.

Exposure with rolling shutter

In experiments involving colloidal particles, the term exposure time typically refers to the user-defined integration time set in the imaging software. However, when using scientific CMOS (sCMOS) cameras—especially in rolling shutter mode—the real temporal window over which particle motion is integrated is more complex. For a moving particle, its image is recorded over a time interval that combines both the set exposure time and the readout delay across the pixel rows it occupies. sCMOS cameras are widely used in high-resolution, high-speed microscopy due to their low readout noise, high quantum efficiency, and fast frame rates. Most sCMOS sensors operate using a rolling shutter mechanism by default, in which rows of pixels are exposed sequentially with a fixed time offset, known as the line time (typically on the order of $10\,\mu$s) [6]. This staggered exposure scheme allows continuous image acquisition with minimal dead time, but it introduces a temporal skew across the frame that becomes relevant for moving objects. Importantly, even when the user-specified exposure time is short, the rolling shutter behaviour typically remains active unless explicitly overridden by a global shutter mode, which is not universally supported. Or it comes with other trade-offs such as lower frame rate and higher noise level. As a result, for fast dynamics or rapidly moving particles, the effective temporal resolution of the image is limited not just by the integration time, but also by the cumulative delay across the imaged region.

To image two colloidal particles confined in optical traps, we employed a scientific sCMOS camera operating in rolling shutter mode. In this configuration, each row of pixels begins and ends its exposure at slightly staggered times, determined by a fixed line readout delay (typically $\sim 10\,\mu$s). Consequently, the effective exposure window

for each particle is not solely determined by the user-defined exposure time, but also by the time required to scan across the vertical extent of the particle. In our setup, the particles span approximately 7 - 10 pixel rows, resulting in an additional temporal offset of up to 70 - 100 μs due to rolling readout.

This offset becomes especially relevant when the exposure time is short—on the order of or smaller than the readout delay across the particle image—where the rolling shutter introduces non-negligible temporal skew across the particle's profile. Under such conditions, the apparent position and shape of the particles may be distorted, particularly if they undergo Brownian motion during acquisition. Conversely, when using longer exposure times (e.g., several milliseconds), the contribution of the line delay to the total integration window becomes negligible. In this regime, the dominant source of temporal averaging is particle diffusion during the exposure, rather than the shutter's readout dynamics.

While the above considerations highlight the role of exposure time and rolling shutter readout in shaping the effective imaging window, it is important to acknowledge that the dynamics observed during exposure are also influenced by the interaction between the particle pair. In particular, the relative motion between two trapped colloidal particles is not only a function of their individual diffusion, but also of hydrodynamic coupling and interparticle forces, which may lead to correlated or constrained motion within the exposure window. Due to these complexities, a purely analytical estimation of motion blur or dynamic averaging is insufficient. Instead, we experimentally quantify the relative motion during exposure using the method described in our previous work [1]. In this approach, all frames corresponding to a specific interparticle distance are selected from the video. The mean relative motion is then calculated as a function of lag time τ by averaging the change in interparticle distance with respect to the initial frame. The dynamic error σ_D is defined as the extrapolated value of this relative motion at τ equal to the exposure time (with readout delay), providing a direct measure of the effective motion blur induced by Brownian dynamics during image acquisition. For simplicity, we continue to refer to the exposure time by the value set in the acquisition software.

The experimentally measured relative motion for different exposure times is plotted in Figure S6. The initial distance range is set to 510–550 nm. As the particle diameter is 500 nm, this range corresponds to close proximity, where hydrodynamic coupling between the particle pair becomes significant. In this regime, the motion of each particle is no longer independent, and the relative displacement reflects both Brownian fluctuations and correlated motion due to the surrounding fluid. By analysing the relative motion as a function of exposure time, we directly capture the degree of dynamic averaging that occurs during image acquisition. In the case of an exposure time of $25\,\mu$s, the relative motion is extrapolated to be approximately 3 - 4 nm (see figure S6 a). However, the readout delay from the rolling shutter (estimated at about $70\,\mu$s) significantly extends the effective time window over which motion is recorded. This results in a total effective exposure time of roughly $100\,\mu$s. Accordingly, we extrapolate the relative motion at 10^{-4} s to be 5 nm and use this value as the dynamic error σ_D for the $25\,\mu$s exposure case in the main text. In contrast, for longer exposure times of $250\,\mu$s and $2500\,\mu$s, the relative contribution of the readout delay is much smaller, and the dynamic error is dominated by Brownian motion during the integration time. From figure S6 b and c, we obtain dynamic errors σ_D of 8 nm (at 3×10^{-4} s) and 12 nm (at 2.5×10^{-3} s) for these two cases, respectively.

References

[1] Chi Zhang, José Muñetón Díaz, Augustin Muster, Diego R Abujetas, Luis S Froufe-Pérez, and Frank Scheffold. Determining intrinsic potentials and validating optical binding forces between colloidal particles using optical tweezers. *Nature Communications*, 15(1):1020, 2024.

[2] Michael M Burns, Jean-Marc Fournier, and Jene A Golovchenko. Optical binding. *Physical Review Letters*, 63(12):1233, 1989.

[3] Augustin Muster, Diego R Abujetas, Frank Scheffold, and Luis S Froufe-Pérez. CoupledElectricMagneticDipoles. jl-julia modules for coupled electric and magnetic dipoles method for light scattering, and optical forces in three dimensions. *Computer Physics Communications*, 306:109361, 2025.

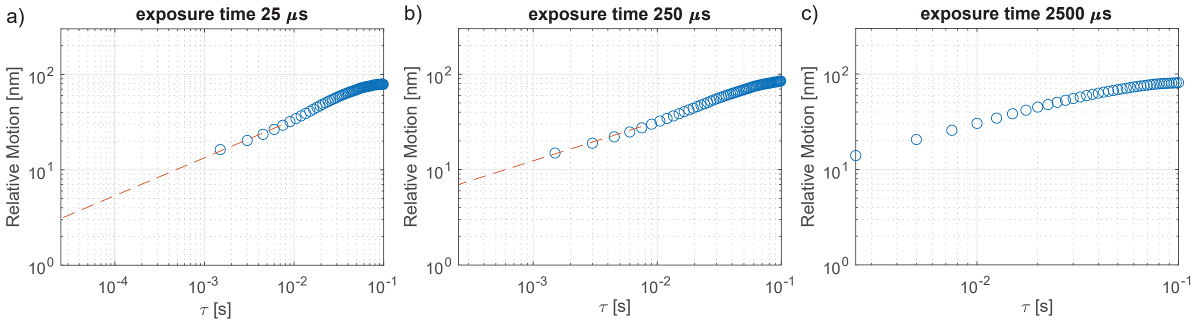

Figure S6: Relative motion of two polystyrene particle of $R = 250$ nm in a line trap, plotted as a function of lag-time τ, for particles with initial distances (the distance at lag-time of 0) in between 510 to 550 nm, for exposure time of $25\,\mu$s (a), $250\,\mu$s (b) and $2500\,\mu$s (c).

[4] Silvia Albaladejo, R Gómez-Medina, Luis S Froufe-Pérez, H Marinchio, R Carminati, JF Torrado, G Armelles, A García-Martín, and Juan José Sáenz. Radiative corrections to the polarizability tensor of an electrically small anisotropic dielectric particle. *Optics Express*, 18(4):3556–3567, 2010.

[5] Sheldon M. Ross. *Stochastic Processes*. Wiley, 2nd edition, 1996.

[6] Teledyne Photometrics. Rolling vs. global shutter.
